# Usnic acid induces apoptosis and inhibits cell migration and invasion in hepatocarcinoma cells: in vitro and in silico analysis

**DOI:** 10.1186/s12906-026-05354-x

**Published:** 2026-03-23

**Authors:** Dogukan Mutlu

**Affiliations:** Department of Molecular Biology and Genetics, Faculty of Engineering and Natural Sciences, Üsküdar University, Istanbul, Turkey

**Keywords:** Usnic acid, Hepatocellular carcinoma, Anticancer, Apoptosis, Antimigration

## Abstract

**Background:**

Lichens produce several secondary metabolites with significant biological and pharmacological activities, among which usnic acid (UA) is particularly notable. This study aimed to investigate the cytotoxic activity, effects on cell migration and invasion, and the mechanisms underlying UA-induced cell death in the human hepatocarcinoma cell line Hep3B.

**Methods:**

Hep3B cells were treated with UA for 24 and 48 h to evaluate its cytotoxicity. Gene expression levels associated with apoptosis and cell migration were analyzed. Functional assays, including scratch-wound and Matrigel invasion assays, were employed to assess migration and invasion. Additionally, molecular docking studies were conducted to elucidate the interactions between UA and potential protein targets.

**Results:**

UA demonstrated significant cytotoxic effects on Hep3B cells, with an IC_50_ of 19.677 µM at 48 h. UA treatment induced apoptosis and effectively suppressed cell migration and invasion. Molecular docking analyses revealed strong binding interactions between UA and key target proteins, as evidenced by negative binding energy values for all complexes.

**Conclusions:**

These findings indicate that UA exerts cytotoxic effects on Hep3B cells by inducing apoptosis and inhibiting migration and invasion. The molecular docking results further suggest UA as a potential lead compound for anticancer drug development. Further investigations focusing on its molecular targets and derivatives are warranted to explore its full therapeutic potential.

**Supplementary Information:**

The online version contains supplementary material available at 10.1186/s12906-026-05354-x.

## Introduction

Cancer remains one of the leading causes of mortality worldwide, fundamentally characterized by the uncontrolled growth and dissemination of abnormal cells [1]. While significant advancements have been achieved in traditional treatment modalities—such as surgery, chemotherapy, radiation, and immunotherapy—many malignancies continue to present challenges due to recurrence, resistance, and systemic toxicity [2, 3]. Consequently, the ongoing development of more effective and precisely targeted therapeutic agents remains a critical priority in oncology. It is in this context that natural compounds with demonstrated anticancer activity continue to attract considerable research interest due to their structural diversity and perceived biocompatibility [4].

Among the various cancer types, Hepatocellular Carcinoma (HCC), the most common form of liver cancer, ranks among the most prevalent and lethal malignancies worldwide. The clinical management of HCC is significantly complicated by its aggressive biological nature and the unfortunate fact that the majority of cases are diagnosed at an advanced stage, limiting curative options [5]. Current standard-of-care therapies, including surgical resection and systemic treatments, often provide limited long-term survival benefits, highlighting a pressing need for novel and less toxic therapeutic strategies to improve patient outcomes. Therefore, this critical therapeutic gap has spurred significant interest in the search for new and potent anticancer agents derived from natural sources [6]. The exploration of natural products as a source for novel anticancer properties has been a pivotal focus in drug discovery for decades. Plant-derived substances, such as paclitaxel and vincristine, have already established the successful precedent for utilizing nature's vast chemical reservoir in clinical chemotherapy [4]. Furthermore, natural products are increasingly valued for their potential advantages over synthetic drugs, including greater perceived biocompatibility and potentially reduced systemic toxicity in comparison to traditional chemotherapeutics [7]. This continuous search for unique bioactive molecules has brought attention to less conventional sources, such as lichens and their unique array of secondary metabolites.

Among the most intriguing natural sources, lichens—complex symbiotic organisms composed of fungi and algae or cyanobacteria—have garnered attention for their distinct secondary metabolites [8]. These secondary metabolites are compounds that are not directly involved in the organism’s fundamental growth processes but play crucial roles in environmental protection, defense, and adaptation. The unique chemical structures of these lichen-derived compounds often translate into a wide range of promising biological activities, including antibacterial, antifungal, and increasingly, anti-proliferative effects [9, 10].

One such metabolite is usnic acid (UA), a well-known lichen-derived compound [11]. UA is particularly notable for its broad spectrum of biological activities and is found primarily in species like *Usnea* and *Cladonia* [12]. UA has been used in various applications, including cosmetics, antibiotics, and traditional medicine for wound healing and skin infections [12–14]. Its antimicrobial properties have made it an attractive compound in the pharmaceutical industry. Recent studies have also shown its potential for other therapeutic uses, including anticancer, anticholinergic, and anti-inflammatory activities [15–17]. Crucially, UA has shown compelling promise as an anticancer agent, demonstrating the ability to inhibit the proliferation of numerous cancer cell lines [18–21]. The anticancer potential of UA is particularly exciting because it can interfere with multiple pathways involved in cancer progression. Research has shown that UA exerts its anticancer effects through several key mechanisms: cell cycle arrest, induction of apoptosis, and modulation of autophagy [19, 20, 22]. However, its specific molecular mechanisms and efficacy in relevant models, such as HCC, still require dedicated investigation.

Building upon the established anticancer potential of UA, this study comprehensively investigated its mechanism of action and efficacy against hepatocellular carcinoma (HCC) using Hep3B cells. The hypothesis tested was that UA inhibits HCC progression by inducing apoptosis and modulating relevant gene expression. The research design employed multiple in vitro assays—including MTT, scratch, flow cytometry, and RT-qPCR—to assess the anti-proliferative, anti-migratory, and apoptotic effects of UA. To evaluate selectivity and potential anti-angiogenic properties, Human Umbilical Vein Endothelial Cells (HUVEC) were included as a critical non-cancerous comparison model. Additionally, molecular docking was utilized to predict direct molecular targets. This research provides a detailed molecular roadmap of UA's inhibitory action against HCC, supporting its consideration as a selective therapeutic agent.

## Material and methods

### Cell culture

Human hepatocellular carcinoma cell line (Hep3B) and normal human umbilical vein endothelial cell line (HUVEC) were purchased from American Type Culture Collection (ATCC). Cells were cultured with Dulbecco’s Modified Eagle’s Medium (DMEM, Sigma-Aldrich, Germany) with 10% fetal bovine serum (FBS, Capricorn, Germany) and 100U/ml of penicillin, and 100 µg/ml of streptomycin (Capricorn) at 37 °C in a humidified 5% CO_2_, as previously described [23].

### Cell viability assay

The effect of UA on Hep3B cell viability was determined using the MTT (3-(4,5-Dimethylthiazol-2-yl)−2,5-Diphenyltetrazolium Bromide) assay as described previously [24]. Briefly, Hep3B and HUVEC cells were plated into 96-well plates (2.5 × 10^3^ cells/well). UA was obtained from Sigma-Aldrich (CAS, # 7562–61-0) was dissolved in chloroform (Sigma-Aldrich) and then various concentrations (3.125, 6.25, 12.5, and 25 µM) of UA were used to treat both cell lines for 24 and 48 h. In all experiments, the final concentration of chloroform was kept below 0.5% (v/v), and the control group was treated with chloroform alone. After treatment periods, the supernatant was evaporated, 10 µl of MTT solution (5 mg/ml, Merck, USA) was added into each well and cells were incubated at 37 °C. After 4 h, the culture medium was removed and 100 µl of dimethyl sulfoxide (DMSO, Carlo Erba, Italy) was added to each well for dissolve the formazan. After 30 min, cell viability was measured at 590 nm using a Epoch microplate spectrophotometer (BioTek, USA). The results are expressed as percentages of the dose group compared to the control group. IC_50_ values was calculated using GraphPad Prism 9 (GraphPad Software, CA, USA).

### Annexin V-FITC/PI double staining

The Annexin V-FITC/PI Apoptosis Kit (E-CK-A211, Elabscience) was used to quantify apoptotic cells, as described previously [25]. Cells were seeded in six-well plates with a density of 3 × 10^4^ cells/well. Then, cells were treated with the IC_50_ dose of UA for 48 h. Hydrogen peroxide (0.2 mM, Sigma-Aldrich) was used as a positive control. After incubation, cells were collected, washed with phosphate buffered saline (PBS, Capricorn) and resuspended in cold Annexin binding buffer. To evaluate the apoptotic cell death, cells were stained with propidium iodide (PI) and Annexin V-FITC. The analysis was performed using a CytoFlex flow cytometer (Beckman Coulter) with in-built CytExpert Software. For each sample, a total of 20,000 events were recorded.

### RNA extraction, cDNA synthesis and RT-qPCR

Hep3B cells (3 × 10^4^ cells) was treated with the IC_50_ dose of UA for 48 h and total RNA was extracted using the innuPREP RNA Mini Kit (Analytik Jena, Germany). cDNA synthesis was performed with 2.5 µg of total RNA using OneScript Plus cDNA Synthesis Kit (ABM, USA). To assess the mRNA levels of *Bax*, *Bcl-2*, *Caspases* (3, 7, 8, and 9*)*, *Bcl-xl*, *PUMA*, *BIM*, *BAK1*, *NOXA*, *VEGF*, *bFGF*, *EGF*, *MMP2*, *MMP9*, *TIMP1*, and *TIMP2*, Real-time quantitative PCR was performed. For RT-qPCR analysis, mRNA expression levels were evaluated using the StepOnePlus™ Real-Time PCR System (Applied Biosystems) with SYBR® Green PCR Master Mix (Applied Biosystems), following the manufacturer’s protocol. The relative gene expression levels were calculated using the 2^−ΔΔCt^ method using GeneGlobe Data Analysis Center (Qiagen), and the control was *β-actin*. The primer sequences are detailed in Supplementary Table 1 and 2.

### In vitro scratch assay

The effect of UA on the motility and the migration ability of Hep3B cells was investigated by the scratch assay, as described previously [26]. Briefly, cells were seeded in 6-well plates (3 × 10^4^ cells/well) and incubated for 24 h. Then, the cell monolayers were scratched with a 200 µl micropipette tip. After scratching, the wells were gently washed with PBS to discard cell debris, and the culture medium was refreshed. Then, Hep3B cells were treated with the IC_50_ dose of UA for 48 h. The wound areas were captured at 0, 24, 48, and 72 h with an inverted microscope (10 × magnification, Oxion Inverso, Euromex, Netherlands) and the closure rate (%) was calculated with ImageJ software 1.53e (USA).

### Invasion assay

Cell invasion assay was performed in 24-well using the Corning Matrigel Invasion Chamber (354,480, BioCoat, USA), as described previously [27]. Briefly, 2 × 10^4^ cells were plated onto the upper chamber in serum-free medium with IC_50_ dose of UA and incubated at 37 °C for 48 h. The cells invaded through Matrigel were stained with crystal violet and captured by inverted microscope.

### Toxicity of UA compared to standard drugs

The toxicity levels of UA and standard drugs were assessed using the online tool ProTox-3.0 (https://tox.charite.de/protox3/) [28]. This platform predicts the LD_50_ (Lethal Dose for 50%) of drugs and UA using mol files. ProTox-3.0 utilizes molecular similarity, fragment propensities, common features, and a machine-learning approach called fragment similarity-based CLUSTER cross-validation, based on a total of 61 models to predict various toxicity endpoints, including adverse outcome pathways (Tox21), metabolism, molecular initiating events, acute toxicity, and organ toxicity.

### Molecular docking studies

Molecular docking of UA with *Caspase-3*, *Caspase-9*, *MMP2*, and *MMP9* was completed using the SeamDock web server (https://bioserv.rpbs.univ-paris-diderot.fr/services/SeamDock/) [29]. Autodock Vina was used to assess protein–ligand interactions with the following settings: grid spacing of 1 Å, energy range of 5 kcal/mol, and exhaustiveness of 8, with the docking box size set to 25 × 25x25 Å. The docking models with high scores were selected. Figures were generated with PyMol v3.0.4, and BIOVIA Discovery Studio v.24.1.0. The interactions between UA and *Caspase-3*, *Caspase-9*, *MMP2*, and *MMP9* were analyzed using Discovery Studio. Additionally, the inhibitor that was bound to *MMP9* was removed prior to the docking studies. The data for the three-dimensional (3D) structures of proteins for *Caspase-3* (PDB ID: 3GJQ), *Caspase-9* (PDB ID: 2AR9), *MMP2* (PDB ID: 1QIB), and *MMP9* (PDB ID: 4H1Q) were used in this study.

### Statistical analysis

GraphPad Prism 9 (San Diego, CA, USA) was used for statistical analysis. The experiments were performed in triplicate, and the data are expressed as means ± SD. Two-way ANOVA was used to analyse the statistical differences between the groups.

## Results

### Effects of UA on Hep3B and HUVEC cell viability

**Fig. 1:**
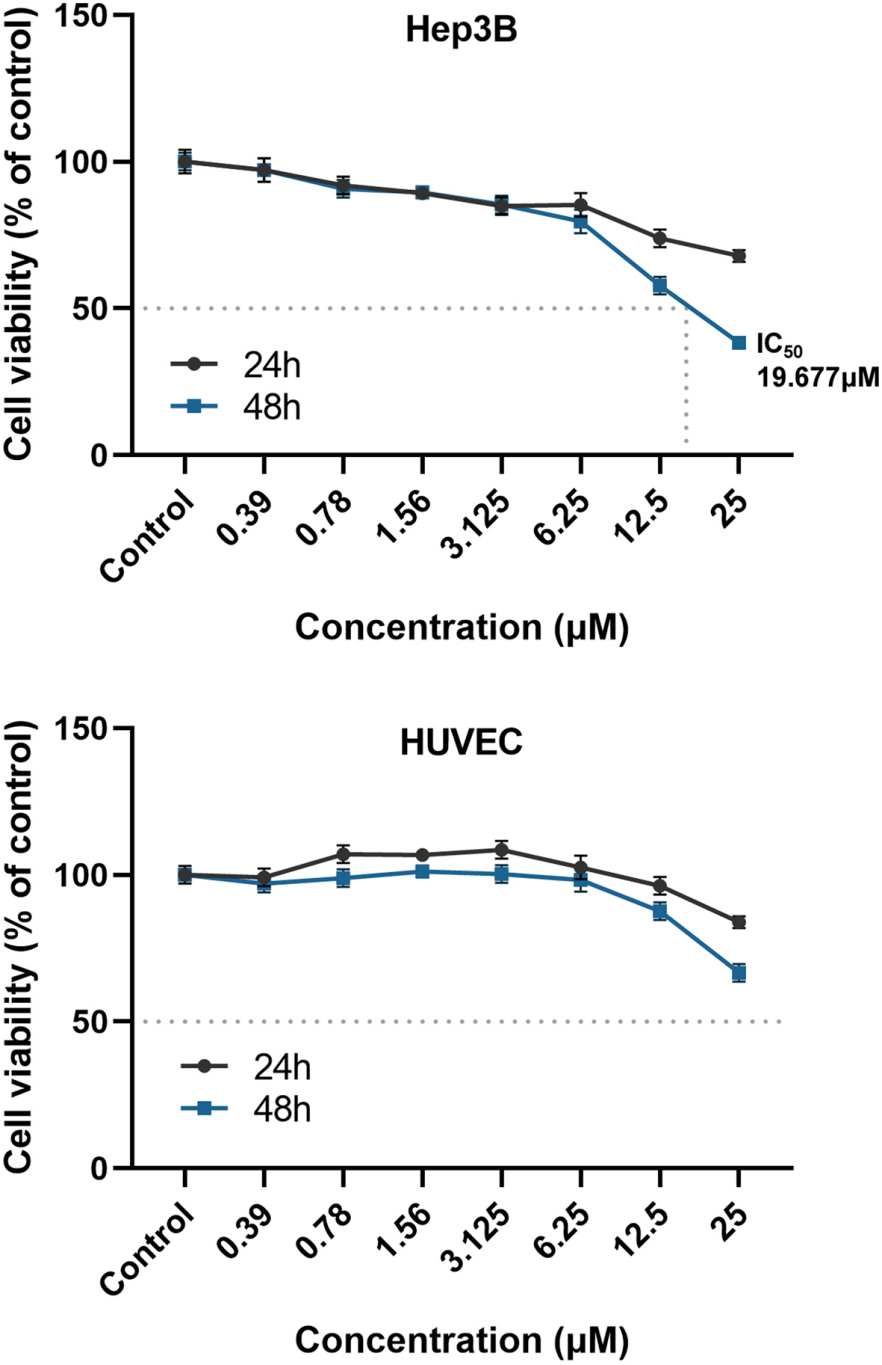
Dose–response curves illustrating the effect of UA on the viability of Hep3B (cancer) and HUVEC (non-cancerous endothelial) cells after 24 h (black line) and 48 h (blue line) of exposure. Cell viability was determined by the MTT assay. Results are expressed as mean ± standard deviation, and the IC_50_ (half-maximal inhibitory concentration) was determined using GraphPad Prism

To investigate the cytotoxic activity, Hep3B and HUVEC cells were treated with different concentrations of UA for 24 and 48 h. The cell viability was measured using MTT reagent. As shown in Fig. 1, UA exhibited cytotoxic activity against Hep3B cells at 48 h, and the IC_50_ value was found to be 19.677 ± 0.978 μM. In contrast, UA was able to reduce the viability of non-cancerous HUVEC cells by 43.4% after 48 h of exposure.

### Flow cytometry analysis

**Fig. 2:**
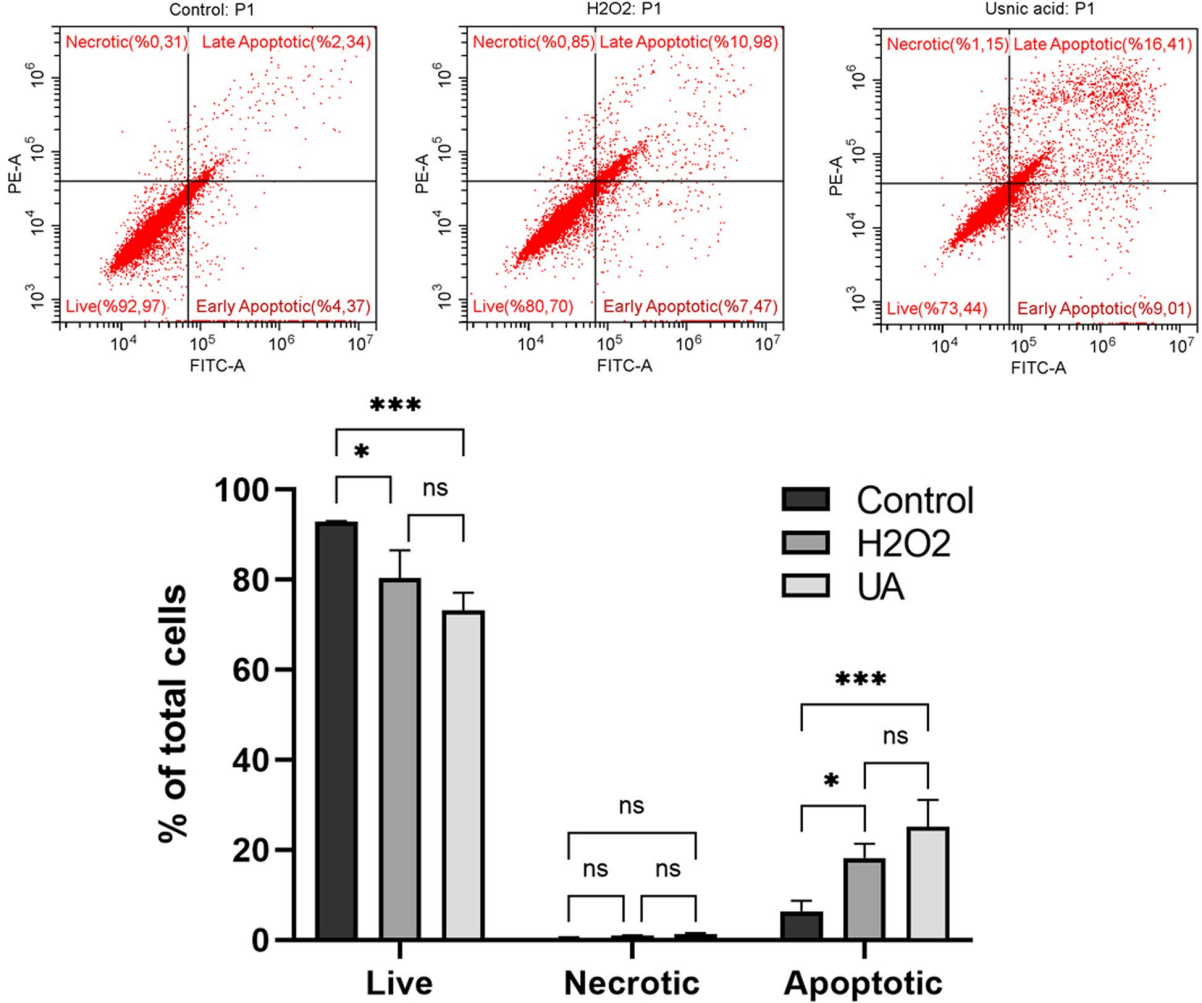
Hep3B cells were treated with the IC_50_ concentration of UA (19.677 µM) for 48 h, and apoptotic cells were quantified using Annexin V-FITC and PI double staining detected by flow cytometry. H2O2 (0.2 mM) was used as a positive control. (Top Panels): Representative scatter plots show the distribution of cells into viable (Annexin V-/PI), early apoptotic (Annexin V +/PI), late apoptotic (Annexin V +/PI), and necrotic (Annexin V-/PI) populations. (Bottom Panel): Bar graphs display the percentage of live, necrotic, and apoptotic cells (early + late apoptosis). ns = no significant, * = *p <* 0.02, *** = *p <* 0.001

As shown in Fig. 2, after 48 h of treatment with UA, 25.42% of the Hep3B cells were apoptotic, 73.44% were alive, and 1.15% were dead. The ratio of apoptotic cells (25.42%) in the UA treatment group was approximately four times higher than in the control group (6.71%). These findings indicate that UA induces apoptosis and exhibits cytotoxic effects. Furthermore, treated cells showed a significant reduction in the percentage of live cells compared to the control group (*p <* 0.001).

### Gene expression analysis

To further clarify the possible mechanism of action of UA, this study focused on the type of cell death in the hepatocarcinoma cell line Hep3B. Previous studies have suggested the involvement of UA in modulating cell death mechanisms. Notably, for the first time, this research demonstrated the expression levels of apoptosis-related genes in Hep3B cells following UA treatment.

Gene expression analysis revealed that UA increased the pro-apoptotic *Bax* (6.82-fold) and decreased the anti-apoptotic *Bcl-2* (17.03-fold) mRNA levels, as summarized in Table 1. Consequently, UA treatment also increased the Bax/Bcl-2 ratio. Moreover, UA caused a 4.45-, 3.33-, and 8.94-fold increase in *Caspase-3*, *Caspase-7*, and *Caspase-8* mRNA levels, respectively.

**Table 1:** Gene expression changes of UA treated Hep3B cells compared to the control group. Real-time qPCR data were analyzed using the QIAGEN GeneGlobe Data Analysis Center, and target genes were normalized to *β-actin*

Gene name	Fold regulation	*p*-value	Gene name	Fold regulation	*p*-value
*Bax*	6.82	0.094255	*VEGF*	0.64	0.140468
*Bcl-2*	−17.03	0.002405	*bFGF*	0.63	0.092526
*Caspase-3*	4.45	0.003462	*EGF*	0.55	0.214972
*Caspase-7*	3.33	0.119892	*MMP2*	6.70	0.374544
*Caspase-8*	8.94	0.032641	*MMP9*	0.13	0.059818
*Caspase-9*	1.92	0.087264	*TIMP1*	3.02	0.000896
*Bcl-xl*	−1.27	0.511139	*TIMP2*	1.21	0.600841
*PUMA*	5.45	0.083209	*β-actin*	1	nan
*BIM*	4.04	0.039238			
*BAK1*	3.01	0.102714			
*NOXA*	1.17	0.512593			

qRT-PCR analysis further indicated that UA suppresses the mRNA levels of genes associated with wound healing. *VEGF* (0.64-fold), *bFGF* (0.63-fold), and *EGF* (0.55-fold) were significantly downregulated. Interestingly, *MMP2* expression increased by 6.70-fold, whereas *MMP9* expression decreased to 0.13-fold. Similarly, *TIMP1* and *TIMP2* were elevated by 3.02- and 1.21-fold, respectively.

### Effects of UA on cell migration and invasion

**Fig. 3:**
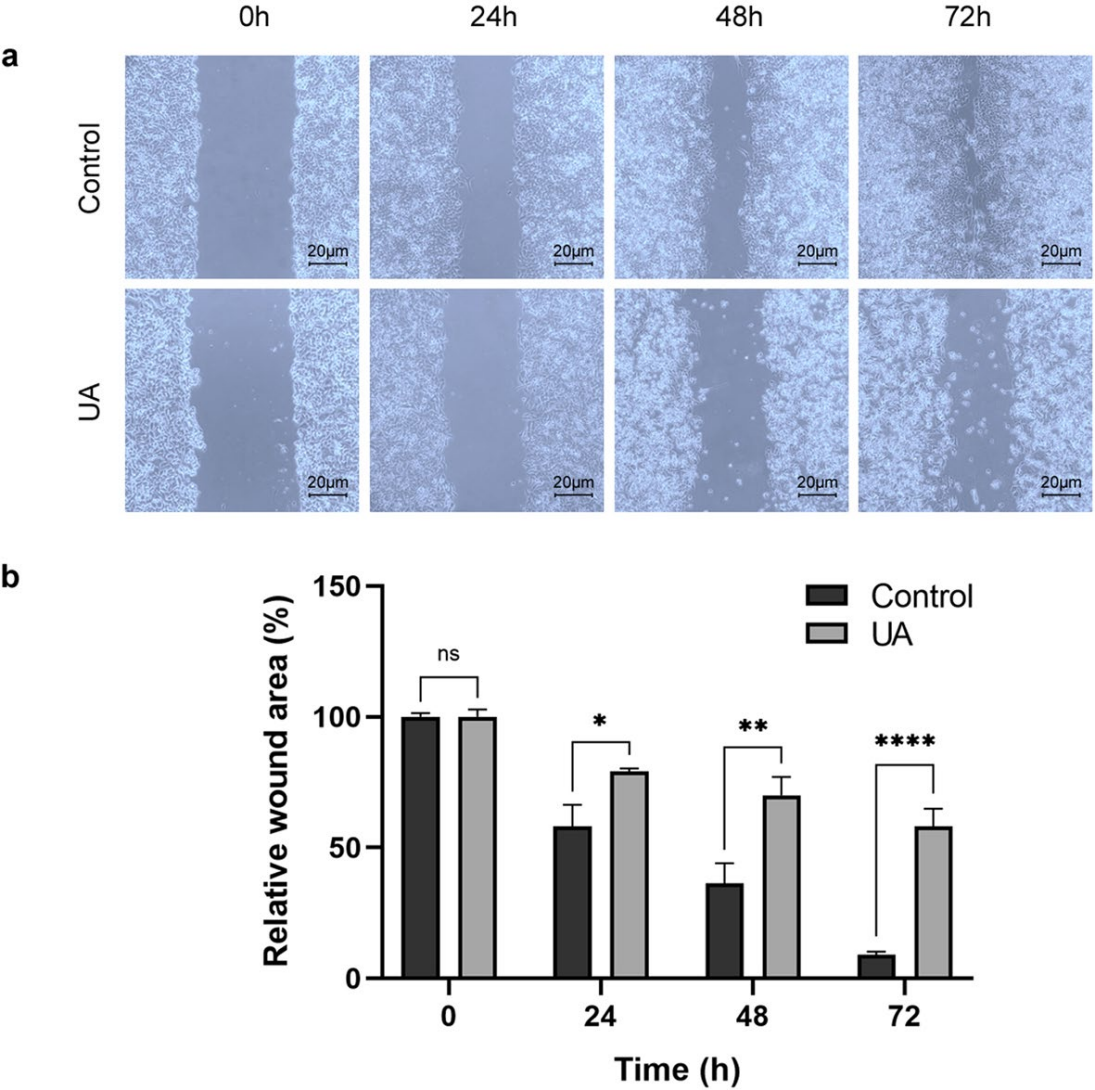
The anti-migratory effect of UA on Hep3B cells was evaluated using an in vitro scratch assay. Cells were treated with the IC_50_ concentration of UA (19.677 µM) following the scratch. **a** Representative images of the wound area closure at 0 h, 24 h, 48 h, and 72 h. **b** Quantification of the relative wound area over time, expressed as a percentage of the initial 0 h area. Data are expressed as the mean ± standard deviation of two independent experiments. ns = no significant, * = *p <* 0.02, ** = *p <* 0.002, **** = *p <* 0.0001

To further examine how UA treatment inhibited cell proliferation, a scratch assay was performed (Fig. 3). The results indicated that control cells nearly filled the wound area (91.71%) by 72 h. On the contrary, treatment of Hep3B cells with UA greatly inhibited their proliferation and motility, and 58.45% of the wound remained unfilled at 72 h (*p <* 0.0001). These results are a further indication that UA treatment of hepatocellular carcinoma cells impairs their ability to proliferate as well as hinders their motility.

**Fig. 4:**
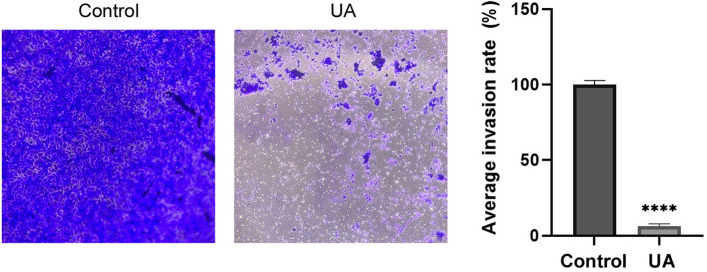
The effect of UA on the invasiveness of Hep3B cells was assessed using the Matrigel transwell invasion assay over 48 h. Cells were treated with the IC_50_ concentration of UA (19.677 µM). Invaded cells were stained with crystal violet and quantified using ImageJ software. The bar graph shows the average invasion rate (%) of Hep3B cells relative to the control group. **** = *p <* 0.0001

Subsequently, an invasion assay was employed to investigate the effects of UA on the migration and invasion abilities of Hep3B cells. As shown in Fig. 4, the crystal violet staining revealed an average 94.13% decrease in the number of invaded Hep3B cells compared with the control group. These results suggest that UA has promising potential to inhibit the invasion of Hep3B cells.

### ProTox-3

Organ and toxicity end points for UA are presented in Table 2. According to Table 2, UA exhibits a relatively low toxicity profile. UA is predicted to be non-hepatotoxic with a probability score of 0.61, while the prediction scores for respiratory toxicity, carcinogenicity, BBB penetration, clinical toxicity, and nutritional toxicity are 0.66, 0.57, 0.71, 0.59, and 0.68, respectively. It is classified as inactive in terms of organ toxicities such as hepatotoxicity, neurotoxicity, and nephrotoxicity, while it is considered active in areas like respiratory and clinical toxicity. Additionally, UA has a 0.57 likelihood of being carcinogenic, suggesting that long-term exposure may carry potential risks. It also appears to be active in affecting mitochondrial membrane potential, which indicates that it could induce apoptosis mechanisms.

**Table 2:** The predicted toxicity of UA by ProTox-3 webserver

Classification	Target	Prediction	Probability
Organ toxicity	Hepatotoxicity	Inactive	0.61
Organ toxicity	Neurotoxicity	Inactive	0.83
Organ toxicity	Nephrotoxicity	Inactive	0.66
Organ toxicity	Respiratory toxicity	Active	0.66
Organ toxicity	Cardiotoxicity	Inactive	0.88
Toxicity end points	Carcinogenicity	Active	0.57
Toxicity end points	Immunotoxicity	Inactive	0.51
Toxicity end points	Mutagenicity	Inactive	0.89
Toxicity end points	Cytotoxicity	Inactive	0.72
Toxicity end points	BBB-barrier	Active	0.71
Toxicity end points	Clinical toxicity	Active	0.59
Toxicity end points	Nutritional toxicity	Active	0.68
Nuclear receptor signaling pathways	Aromatase	Inactive	0.74
Stress response pathways	Mitochondrial Membrane Potential (MMP)	Active	0.64
Stress response pathways	Phosphoprotein (Tumor Supressor) p53	Inactive	0.58

### Interaction analysis

**Fig. 5:**
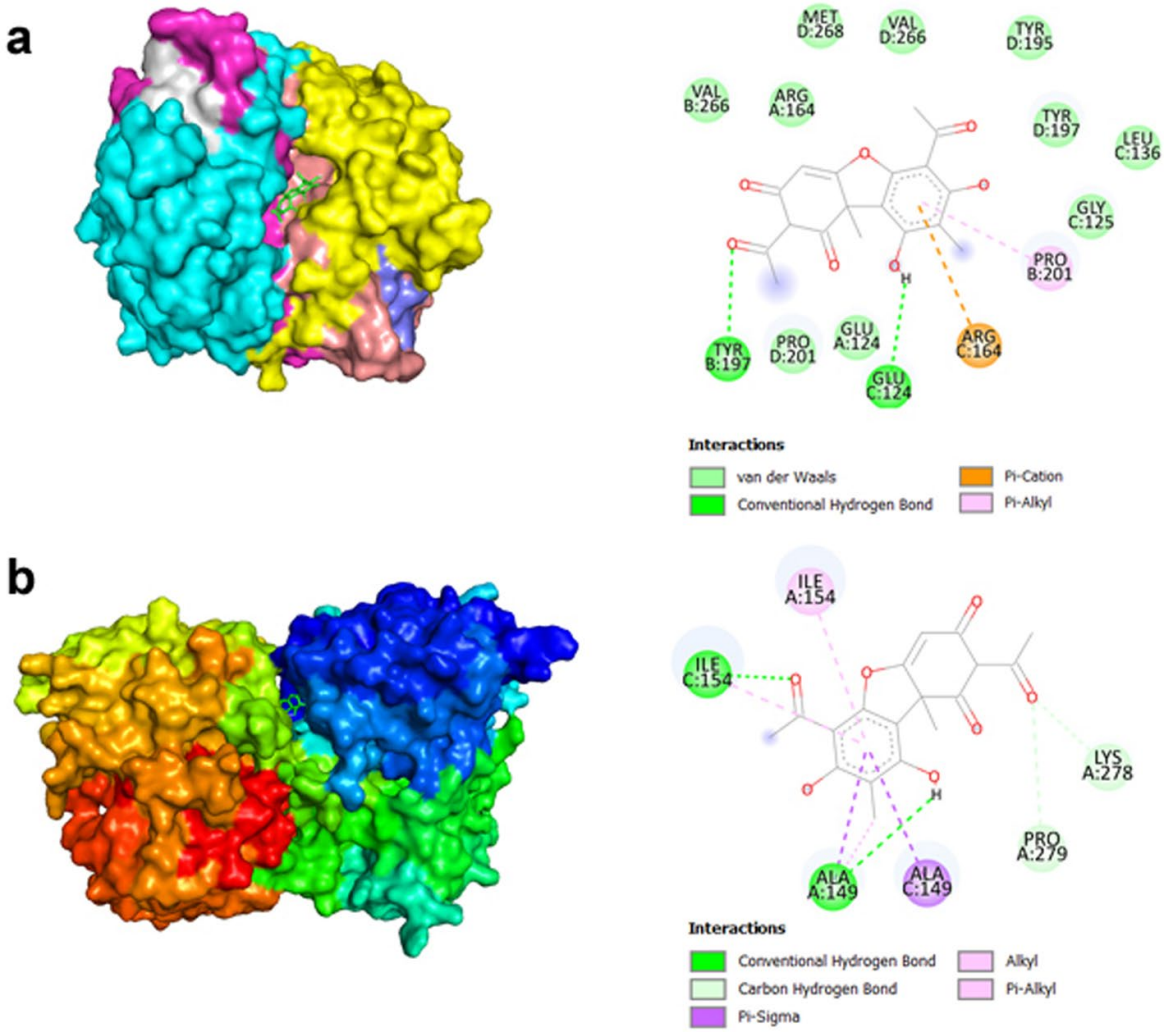
Molecular docking analysis showing the predicted binding patterns of UA (ligand) within the active sites of (**a**) *Caspase-3* and (**b**) *Caspase-9* (receptor proteins), whose 3D structures were obtained from the PDB database (Caspase-3: 3GJQ, Caspase-9: 2AR9)

**Fig. 6:**
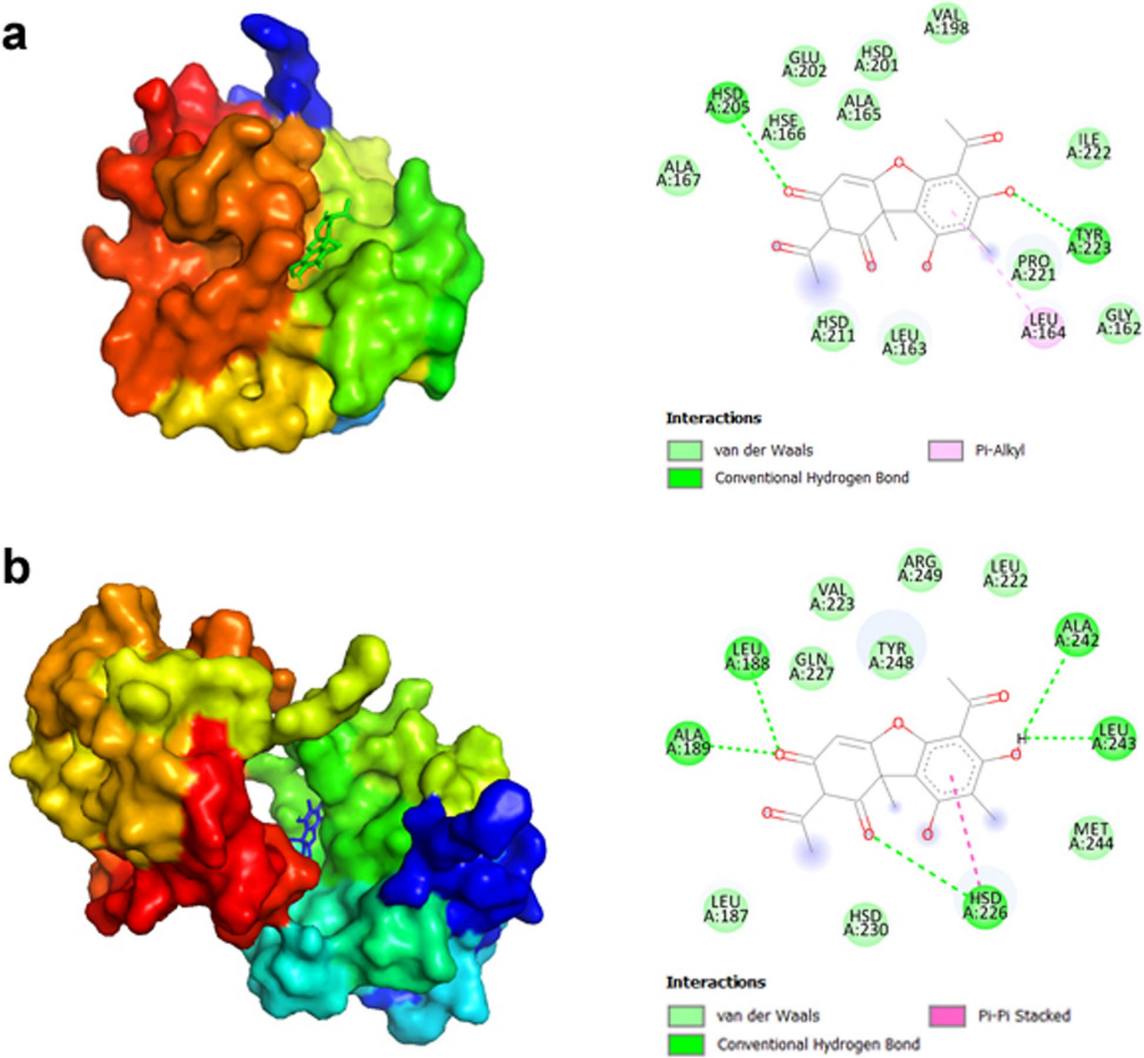
Molecular docking analysis showing the predicted binding patterns of UA (ligand) within the active sites of (**a**) *MMP-2* and (**b**) *MMP-9* (receptor proteins), whose 3D structures were obtained from the PDB database (MMP2: 1QIB, MMP9: 4H1Q)

The 3D structures of *Caspase-3* and *Caspase-9* were obtained from the PDB database as receptor proteins. The SMILES code of UA (CID: 5646) was obtained from PubChem. UA's potential binding interactions with the amino acids in the active sites of these caspases, as well as matrix metalloproteinases involved in hepatocellular carcinoma, were investigated through molecular docking studies (Figs. 5 and 6).

As shown in Fig. 5, UA is predicted to form interactions with TYR197, GLU124, ARG164, and PRO201 amino acids through hydrogen bonds with the *Caspase-3* protein, with a binding affinity of − 7.9 kcal/mol in the best pose (Table 3). Similarly, for the *Caspase-9* protein, UA, with a binding affinity of − 7.9 kcal/mol, is predicted to interact with ILE154, ALA149, LYS278, and PRO279 amino acids through hydrophobic interactions and hydrogen bonds in the active site.

**Table 3:** Binding affinity of caspases and MMPs with UA

Ligand	Receptor	Binding Affinity (kcal/mol)	Interacting Amino Acids
UA	*Caspase-3*	−7.9	TYR197, GLU124, ARG164, PRO201
UA	*Caspase-9*	−7.9	ILE154, ALA149, LYS278, PRO279
UA	*MMP2*	−6.9	TYR223, LEU164, HSD205
UA	*MMP9*	−7.3	LEU188, ALA189, HSD226, ALA242, LEU243

UA exhibited a predicted potential for interactions with the *MMP2* protein, showing a binding affinity of − 6.9 kcal/mol in the best pose (Table 3). UA is predicted to interact with HSD205 and TYR223 and hydrophobic interactions with LEU164 (Fig. 6). Moreover, for the *MMP9* protein, UA, with a binding affinity of − 7.3 kcal/mol, is predicted to interact with LEU188, ALA189, ALA242, and LEU243 through hydrophobic interactions and with HSD226 through hydrogen bonds in the active pockets.

## Discussion

The primary finding of this study is the potent and selective cytotoxic effect of UA on Hep3B hepatocellular carcinoma cells. At 48 h, UA achieved an IC50 of 19.677 µM in Hep3B cells, whereas the non-cancerous HUVEC endothelial cell line retained > 50% viability at 25 µM, confirming a favorable therapeutic index and selective cytotoxicity. This selectivity aligns with observations by Eryilmaz et al., who similarly found minimal effects of UA on normal cells at concentrations toxic to breast and prostate cancer lines [30]. Mechanistically, UA was shown to induce apoptosis in Hep3B cells, providing a parallel to previous findings in other HCC models. For instance, Yurdacan et al. reported that UA induces G_0_/G_1_ and G_2_/M cell cycle arrest and simultaneous apoptosis and autophagy in HepG2 cells, with an increased Bax/Bcl-2 ratio and *Caspase-3* activation, supporting the present observations [31]. More broadly, these findings agree with previously published research demonstrating the anticancer properties of UA against various cancer types, including non-small cell lung cancer cells [18], hepatocarcinoma [30], breast [31], prostate [32], skin [17], gastric [33], cervical [34], and colorectal cancer cells [35]. UA’s efficacy in the p53-deficient Hep3B model further highlights the study’s significance by demonstrating its potential to target cancer cells regardless of p53 status.

Beyond cell death, UA effectively impairs tumour cell motility and neovascularization. Scratch and transwell assays (Figs. 3 and 4) revealed potent inhibition of Hep3B migration and invasion. The magnitude of this reduction suggests that UA possesses significant anti-metastatic potential, indicating its ability to interfere with the key processes of tumor spread and aggressiveness. Concurrently, transcriptional profiling in this study showed downregulation of *VEGF*, *bFGF*, *EGF* and altered MMP/TIMP balance (Table 1), consistent with Song et al., who demonstrated that UA inhibits breast tumour angiogenesis in vivo via suppression of VEGFR-mediated AKT and ERK1/2 signaling [36]. These modulations indicate that UA interferes with extracellular matrix remodeling and angiogenesis, processes essential for tumor progression. Since tumor growth depends on angiogenesis, targeting this process is a viable strategy for combating cancer. Overall, these results indicate that UA substantially impairs cell migration by affecting both cell proliferation and extracellular matrix dynamics.

Despite providing a comprehensive in vitro mechanistic assessment of UA in Hep3B cells, this study has several limitations that affect the final interpretation of the findings. Firstly, while HUVEC cells were used to confirm UA's selective cytotoxicity, corresponding migration and invasion assays were not performed on this non-tumor model. Future research must prioritize this comparison to fully differentiate UA's anti-metastatic activity from its potential anti-angiogenic effects. Secondly, the proposed molecular mechanism, which includes Caspase-3/9 and MMP-2/9 targeting, relies solely on computational docking data. While the binding affinities are thermodynamically favorable, the biological confirmation of these interactions requires further rigorous experimental validation, such as co-immunoprecipitation or site-directed mutagenesis studies, to definitively confirm UA’s mechanism of action in vivo. These necessary future steps are essential for translating the current in vitro findings into a reliable clinical context.

## Conclusion

The UA examined in this study showed minimal cytotoxicity towards the HUVEC endothelial cell line and demonstrated notable effectiveness against the Hep3B hepatocarcinoma cell line. Furthermore, it induced apoptosis in a p53-deficient model and inhibited migration and invasion. Coupled with favorable molecular docking predictions suggesting its potential interaction with key apoptotic and metastatic targets, these findings collectively suggest that UA is a promising candidate for the development of safe and effective anticancer agents, particularly for tumors with p53 mutations or high metastatic potential.

## Supplementary Information

Supplementary Material 1.

## Data Availability

No datasets were generated or analysed during the current study.

## Abbreviations

HCC: Hepatocellular carcinoma
cDNA: Complementary DNA
FBS: Fetal bovine serum
MTT: 3-(4,5-Dimethylthiazol-2-yl)-2,5-Diphenyltetrazolium Bromide
HUVEC: Human umbilical vein endothelial cell line
Hep3B: Human hepatocarcinoma cell line
DMEM: Dulbecco’s Modified Eagle’s Medium
ATCC: American Type Culture Collection
PI: Propidium iodide
UA: Usnic acid
DMSO: Dimethyl sulfoxide
IC_50_: Half-maximal inhibitory concentration
H_2_O_2_: Hydrogen peroxide
PBS: Phosphate buffered saline
LD50: Lethal dose, 50%
Bax: Bcl-2-associated X protein
Bcl-2: B-cell lymphoma 2
Bcl-xl: B-cell lymphoma-extra large
PUMA: P53 up-regulated modulator of apoptosis
BIM: Bcl-2 interacting mediator of cell death
BAK1: Bcl-2 homologous antagonist killer
NOXA: Phorbol-12-Myristate-13-Acetate-Induced Protein 1
VEGF: Vascular endothelial growth factor
bFGF: Basic fibroblast growth factor
EGF: Epidermal growth factor
MMPs: Matrix metalloproteinases
TIMPs: Tissue inhibitor of matrix metalloproteinases
