# Supplementary material for: Usnic acid induces apoptosis and inhibits cell migration and invasion in hepatocarcinoma cells: in vitro and in silico analysis
Source: BMC Complement Med Ther. 2026 Mar 23;26:162. doi: 10.1186/s12906-026-05354-x (PMC13130566; doi:10.1186/s12906-026-05354-x)
Supplement: Supplementary file 1 — Supplementary Material 1. [file 12906_2026_5354_MOESM1_ESM.docx]

**Table S1.** Primers for real-time PCR.

| Gene | Primer | Sequence (5′–3′) |
| --- | --- | --- |
| Bax | Forward  Reverse | TCAGGATGCGTCCACCAAGAAG TGTGTCCACGGCGGCAATCATC |
| Bcl-2 | Forward  Reverse | ATCGCCCTGTGGATGACTGAGT GCCAGGAGAAATCAAACAGAGGC |
| Bcl-xl | Forward  Reverse | GCCACTTACCTGAATGACCACC  AACCAGCGGTTGAAGCGTTCCT |
| Caspase-3 | Forward  Reverse | GGAAGCGAATCAATGGACTCTGG  GCATCGACATCTGTACCAGACC |
| Caspase-7 | Forward  Reverse | CGGAACAGACAAAGATGCCGAG  AGGCGGCATTTGTATGGTCCTC |
| Caspase-8 | Forward  Reverse | AGAAGAGGGTCATCCTGGGAGA  TCAGGACTTCCTTCAAGGCTGC |
| Caspase-9 | Forward  Reverse | GTTTGAGGACCTTCGACCAGCT  CAACGTACCAGGAGCCACTCTT |
| PUMA | Forward  Reverse | ACGACCTCAACGCACAGTACGA  CCTAATTGGGCTCCATCTCGGG |
| BIM | Forward  Reverse | CAAGAGTTGCGGCGTATTGGAG  ACACCAGGCGGACAATGTAACG |
| BAK1 | Forward  Reverse | TTACCGCCATCAGCAGGAACAG  GGAACTCTGAGTCATAGCGTCG |
| NOXA | Forward  Reverse | CTGGAAGTCGAGTGTGCTACTC TGAAGGAGTCCCCTCATGCAAG |
| β-actin | Forward  Reverse | CACCATTGGCAATGAGCGGTTC  AGGTCTTTGCGGATGTCCACGT |

**Table S2.** Primers for real-time PCR.

| Gene | Primer | Sequence (5′–3′) |
| --- | --- | --- |
| VEGF | Forward  Reverse | TTGCCTTGCTGCTCTACCTCCA  GATGGCAGTAGCTGCGCTGATA |
| BFGF | Forward  Reverse | AGCGGCTGTACTGCAAAAACGG  CCTTTGATAGACACAACTCCTCTC |
| EGF | Forward  Reverse | TGCGATGCCAAGCAGTCTGTGA  GCATAGCCCAATCTGAGAACCAC |
| MMP2 | Forward  Reverse | AGCGAGTGGATGCCGCCTTTAA  CATTCCAGGCATCTGCGATGAG |
| MMP9 | Forward  Reverse | GCCACTACTGTGCCTTTGAGTC  CCCTCAGAGAATCGCCAGTACT |
| TIMP1 | Forward  Reverse | GGAGAGTGTCTGCGGATACTTC  GCAGGTAGTGATGTGCAAGAGTC |
| TIMP2 | Forward  Reverse | ACCCTCTGTGACTTCATCGTGC  GGAGATGTAGCACGGGATCATG |
